# Simulation acceleration for transmittance of electromagnetic waves in 2D slit arrays using deep learning

**DOI:** 10.1038/s41598-020-67545-x

**Published:** 2020-06-29

**Authors:** Wonsuk Kim, Junhee Seok

**Affiliations:** School of Electrical Engineering, Korea University, Seoul, 02841 South Korea

**Keywords:** Optics and photonics, Optical materials and structures, Mathematics and computing, Computational science

## Abstract

When designing new optical devices, many simulations must be conducted to determine the optimal design parameters. Therefore, fast and accurate simulations are essential for designing optical devices. In this work, we introduce a deep learning approach that accelerates a simulator solving frequency-domain Maxwell equations. Our model achieves high accuracy while predicting transmittance per wavelength in 2D slit arrays under certain conditions to achieve 160,000 times faster results than the simulator. We generated a dataset using an open-source simulator and compared its performance with those of other machine learning models. Additionally, we propose a new loss function and performance evaluation method for creating better performance models with multiple regression outputs from one input source. We observed that using a loss function that adds binary cross-entropy loss, which predicts whether the differential of the transmittance is positive or negative at wavelengths adjacent to the root mean-squared error of the transmittance value, is more effective for predicting variations in multiple regression outputs. The simulation results show that a four-layer convolutional neural network model demonstrates the best accuracy (R^2^ score: 0.86). The overall approach presented here is expected to be useful for simulating and designing optical devices.

## Introduction

Over the past few years, convolutional neural networks (CNNs) have revolutionised the way we solve image classification problems^1, 2^. Owing to their advantages, such as feature learning and high computational efficiency, CNNs are used in various computer vision tasks.

CNNs constitute the major architecture behind the popular object detection models, including region-based convolutional neural networks (R-CNN)^3^, fast R-CNN^4^, and faster R-CNN^5^. They are also used in object tracking^6^, object recognition^7^, and semantic segmentation^8^ to improve the performance of the computer vision approaches. As the performance of each task achieves greater similarity to that of the human eye, many studies are conducted to improve not only the performance but also computational efficiency. Therefore, researchers have developed CNN-based models that dramatically reduce the amount of computations^9, 10^ and have applied these models to mobile devices to demonstrate that they work well with limited computing power^11^.

CNNs also have many novel applications besides computer vision, such as natural language processing^12^, and speech recognition^13^. They outperform traditional densely connected neural networks, for example, in predicting eigenvalue problems in mechanics^14^, automatic feature selection and market prediction in finance^15^, and classifying single cells in thin blood smears on standard microscope slides based on characteristics such as infected or uninfected objects^16^.

An emerging application of CNN is physics-informed deep learning^17^, which is a technique for solving supervised learning tasks while respecting a given law of physics described by its general nonlinear partial differential equations. CNN is suitable in tasks that can display inputs in image form, exhibiting excellent performance^18, 19^. Many researchers have attempted to solve the problem of accurately and quickly estimating partial differential equations that take considerable time to solve, such as Burgers’ equation, Navier–Stokes equation^20^, and Maxwell’s equations^21^.

One of the areas in which simulators are used to solve partial differential equations (PDEs) is computational electromagnetics, where researchers use simulations for antenna and circuit designs, target detection, and nano-optics^22^. The algorithm of the simulation solves Maxwell’s equations under various material and boundary conditions. Many simulators have been developed for solving these algorithms, for example, gprMax^23^ in the finite-difference time-domain (FDTD) method and MaxwellFDFD^21^ in the finite-difference frequency-domain (FDFD) method.

Several approaches have been considered to accelerate these simulations. With the development of hardware-accelerated scientific computing capability provided by graphics processing units (GPUs), both FDTD- and FDFD-based algorithms can be solved with a speed-up factor of more than 20 on a GPU compared to the solution on a central processing unit (CPU)^24–26^. In addition, with the development of deep learning techniques, data-driven acceleration has been garnering attention. Considerable amounts of data have provided new opportunities for data-driven discovery of hidden physical laws. The data-driven model exhibits accurate performance and remarkably efficient predictions in photonic simulations^27–29^ and solving other PDEs^30^.

This study investigates the feasibility of using deep learning and machine learning techniques for accelerating electromagnetic simulations, especially to solve Maxwell’s equations. We present a framework using CNN for fast prediction of transmittance from a designed shape of a material. We generate a dataset through MaxwellFDFD, which solves the FDFD Maxwell’s equation. Then, we use the data to train and evaluate prediction models. We also suggest practical improvements and evaluations of the prediction models using other loss functions and assessment methods.

## Results and discussion

In this section, we present the experimental results. Our experiments comprised assessing predictive performances of various models, running time comparisons between CNN and MaxwellFDFD simulators, and predicting results of the CNN model for several loss functions.

### Performance benchmarks

We trained several machine learning models, including multilayer perceptron (MLP) and CNN, with 27,000 training sets, and measured their performance by calculating the root mean-squared error (RMSE) and R^2^ scores on the validation set. The results for each model are shown in Table 1. Although the input dimensions of the MLP and CNN were 100 times greater than those of the other models, CNN performed the best in the validation set. The extra tree model showed the best performance in the training set, but as seen from the performance in the validation set, it was overfitted. The MLP model also performed sufficiently well.

**Table 1:** Root mean squared errors and R^2^ scores of the validation set by using regression models.

Model	RMSE		R^2^ score	
	Train	Validation	Train	Validation
Convolutional neural network (CNN)	0.0364	0.0623	0.9496	0.8594
Multilayer perceptron (MLP)	0.0630	0.0845	0.8485	0.7411
Random forest	0.0416	0.0772	0.9372	0.7825
Extra tree	0.0292	0.0844	0.9693	0.7418

Extra Tree best-performed in the training set, but CNN performed the best in the validation set.

**Figure 1:**
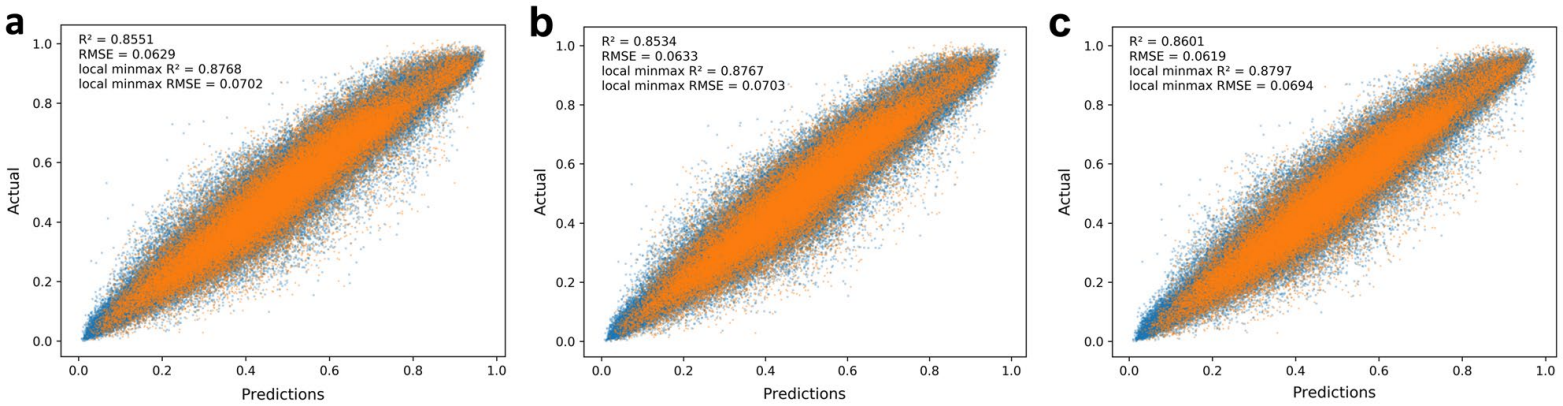
Model performance results on the test set. Estimation of the (**a**) model trained by loss function of RMSE, (**b**) model trained by the loss function that adds the RMSE of the differential to the RMSE, and (**c**) model trained by the loss function that adds the BCE of the differential to the RMSE. The data from the local maxima and minima values and the remaining data are represented by orange and blue dots, respectively.

Because the prediction model used all 24 output transmittances to evaluate performance, it produced smaller fluctuations than the output of the actual simulation. Therefore, we used the aforementioned loss functions to create a realistic model that followed the actual fluctuations, and modified the evaluation method accordingly. More details can be found in ‘Developing a model with practical performance’ section in “Methods and materials” section. The training curves of the networks corresponding to the training set as well as the validation set are plotted in Supplementary Figure S2. The model trained by RMSE and binary cross-entropy (BCE) loss of the differential value of the transmittance of the adjacent wavelength (diff. BCE) as the loss function performed the best in the test set, as shown in Table 2 and Fig. 1. On the other hand, the model trained by RMSE and RMSE of the differential value of transmittance of adjacent wavelengths (diff. RMSE) showed the best performance on the training set among the three models using CNN. To evaluate the performance more practically, the RMSE and R^2^ scores were calculated only from the local maxima and minima values among the results, as demonstrated in Table 3. Even with this evaluation method, the model trained by the loss function that added the BCE of the differential to the RMSE performed the best. In addition, the developed model for Type 2 dataset outperforms the model for Type 1. As illustrated in Supplementary Table S5, the model ‘Type 2’, which is trained and tested with Type 2 dataset, performed better than the model ‘Type 1’. However, as shown by comparing ‘Type 1’ and ‘Type 2-half’, the model trained and tested with the same number of different types of dataset showed similar performance. The model ‘Type 2-half’ performed even worse than the model ‘Type 1’ when tested with local maxima and minima. Therefore, we can observe that developed model for Type 2 does a better job than for Type 1 because it includes more Type 2 dataset.

**Table 2:** Performance comparison between the CNN models in terms of the composition of the loss function.

Model	Loss function	RMSE		R^2^ score	
		Train	Test	Train	Test
CNN	RMSE	0.0364	0.0629	0.9496	0.8551
	RMSE + diff. RMSE	0.0349	0.0633	0.9542	0.8534
	RMSE + diff. BCE	0.0370	0.0619	0.9480	0.8601
MLP	RMSE	0.0630	0.0837	0.8485	0.7438
Random forest	RMSE	0.0416	0.0807	0.9372	0.7605
Extra tree	RMSE	0.0292	0.0880	0.9693	0.7178

Root mean squared errors and R^2^ scores were measured from the overall output values of the train set and test set.

**Table 3:** Performance comparison between the CNN models in terms of the composition of the loss function.

Model	Loss function	RMSE of local maxima and minima		R^2^ score of local maxima and minima	
		Train	Test	Train	Test
CNN	RMSE	0.0407	0.0702	0.9589	0.8768
	RMSE + diff. RMSE	0.0370	0.0703	0.9661	0.8767
	RMSE + diff. BCE	0.0413	0.0694	0.9576	0.8797
MLP	RMSE	0.0709	0.0914	0.8753	0.7913
Random forest	RMSE	0.0431	0.0894	0.9539	0.8006
Extra tree	RMSE	0.0288	0.0954	0.9794	0.7727

Root mean squared errors and R^2^ scores were measured from local maxima and local minima of datasets.

To further improve the performance of the prediction model, we used the ensemble method, i.e. a technique that creates multiple outputs from multiple models and then combines them to attain improved results. The models used for the ensemble method include the aforementioned three models and the model trained by the loss function of the mean-squared error (MSE). The proposed ensemble model was generated by calculating the average of each model’s output. As illustrated in Supplementary Table S4, increasing the number of models improves the performance.

### Execution time

The MaxwellFDFD simulator requires an extended duration of time for operation, while our CNN model needs very little time (Table 4) because the MaxwellFDFD simulator calculates the electric field of each grid and obtains the transmittance. We measured the running time using 9,000 samples in the test set and 27,000 samples in the training set. The execution time per sample was calculated by dividing the time taken in each dataset by the number of samples. Our CNN model was 160,000 times faster with the GPU and 48,000 times faster without the GPU at calculating the transmittance per 24 wavelengths. The execution time was calculated using an NVIDIA GTX 1080Ti GPU and a single 4-core CPU. In addition, it took ~ 3 h for 300 epochs using the same PC to train the model. As shown in Supplementary Fig. S3, the training time of the model with diff. BCE and diff. RMSE differed by 0.1 and 0.22 s per epochs and by 32 and 66 s when performing 300 epochs, respectively, compared to the model using only RMSE. Since the difference in calculation time is less than 3%, which is only about a minute when performing the whole training process, it would be reasonable to use the model using the loss function with diff. BCE added.

**Table 4:** The average execution time comparison between the MaxwellFDFD simulator and the CNN model.

# of samples	MaxwellFDFD	CNN	
		GPU (s)	Non-GPU (s)
1	60 s	0.0004	0.0013
9,000	150 h	3.3	11.3
27,000	450 h	10	34

The execution time was calculated during the Training set (27,000 samples) and the Test set (9,000 samples). CNN model was approximately 160,000 times faster than the simulator when using the graphics processing unit (GPU) and 48,000 times faster without GPU.

### Examples

**Figure 2:**
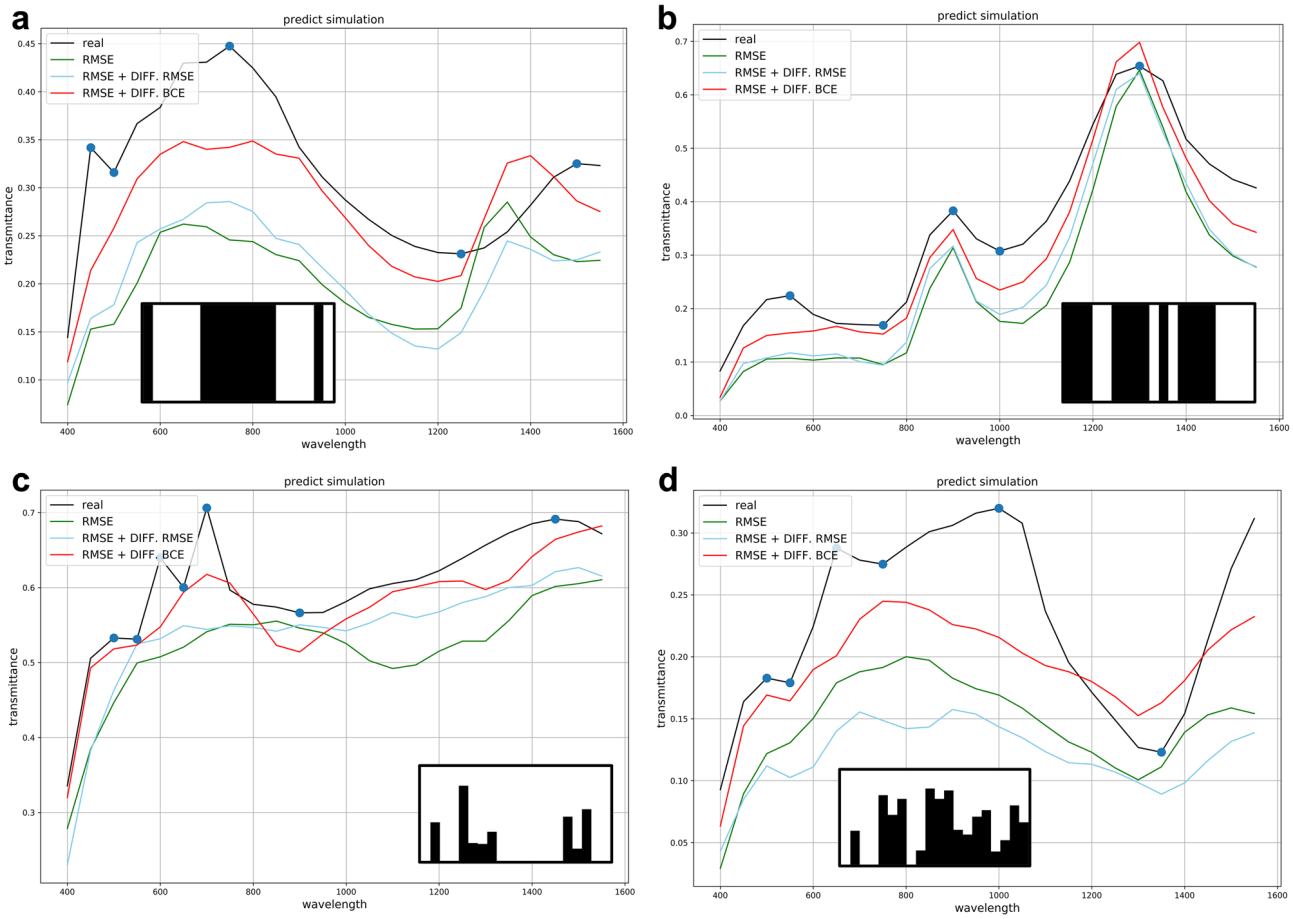
Example prediction results for Type 1 and Type 2 datasets. (**a**) and (**b**) are samples from the Type 1 dataset. (**c**) and (**d**) are samples from the Type 2 dataset. (**a**) Shows relatively high transmittances at low wavelengths, and (**b**) Shows relatively high transmittances at specific wavelengths. (**c**) Shows relatively high transmittances at specific low and high wavelengths, and (**d**) shows relatively high transmittances at medium and high wavelengths. The blue dots indicate the local maxima and local minima, and the input design is shown at the bottom.

Figure 2a, b show some examples of the results among the test sets of Type 1, while Fig. 2c, d show examples from Type 2. The model using the loss function with the BCE of the differential value added was closer to both trends and quantities with real values. For this reason, the model performed the best in predicting not only transmittances among the test sets but also wavelengths that result in local maxima and minima, as indicated by blue dots in Fig. 2.

If we simply set the RMSE of all values to be a performance metric of our model, we would not need a new loss function. As can be seen in Fig. 2, the model with only RMSE as a loss function cannot predict the transmittance curve well to reduce the mean error. Thus, to obtain this as a benchmark for performance evaluation, the RMSEs of the local maxima and minima were calculated, as presented in Table 3. Even though the ratio of RMSE to diff. BCE was not optimised for the best performance, the loss function of the RMSE with the added differential of BCE of the same proportion performed approximately 1 ~ 2% better than usual.

In general image processing, CNN has better performance than MLP, but herein, there was little difference. This is because the input dimension was small, as we used a binary image rather than an RGB colour image. If we expand this model to use other materials instead of silver or a three-dimensional image rather than a two-dimensional image, we can add a physics-informed neuron to a fully connected layer or add a dimension such as that of an RGB colour image. In addition, because of the small input dimension, we designed a shallower layer of CNN.

## Conclusion

We proposed a deep learning approach that accelerates the MaxwellFDFD simulator to calculate the transmittance 160,000 times faster. Fast prediction of transmittance is an important part of optical device design because the designers use simulators for repeating the device design and transmittance prediction processes hundreds of times. Therefore, owing to the reduced computational time, the proposed model allows for more simulation, which makes it possible to achieve the desired performance. By using the new loss function, we not only reduced the computational time but also presented a new approach for improving the performance of multiple regression outputs from an image. The model with the new loss function was better at predicting the local maxima and minima of the output values, as compared to the previous models. Since physical properties of design (e.g., the number of slits or the distance between slits) is essential for the calculation of transmittance, our model can be further improved by combining additional physical quantities in the same network. Similar approaches^18, 31^ have been made in recent work to improve prediction accuracy. In addition, training a model in which the design image is output by substituting the desired transmittance by using generative adversarial networks^32^ or training a model with three-dimensional images will be the objective of the future studies. Furthermore, we expect to apply this method to density functional theory (DFT) calculations used to predict electronic structures^33^ or to apply other deep learning methods, such as recurrent neural networks^34^, for the future work.

## Methods and materials

Our method aims to train a machine-learning model for predicting the transmittance from a designed shape of a material. It comprises the following sections, generating dataset, training model, evaluating the performance, and developing a model with more practical performance, which can predict the local maxima and minima of the transmittance more accurately.

### Generating dataset

**Figure 3:**
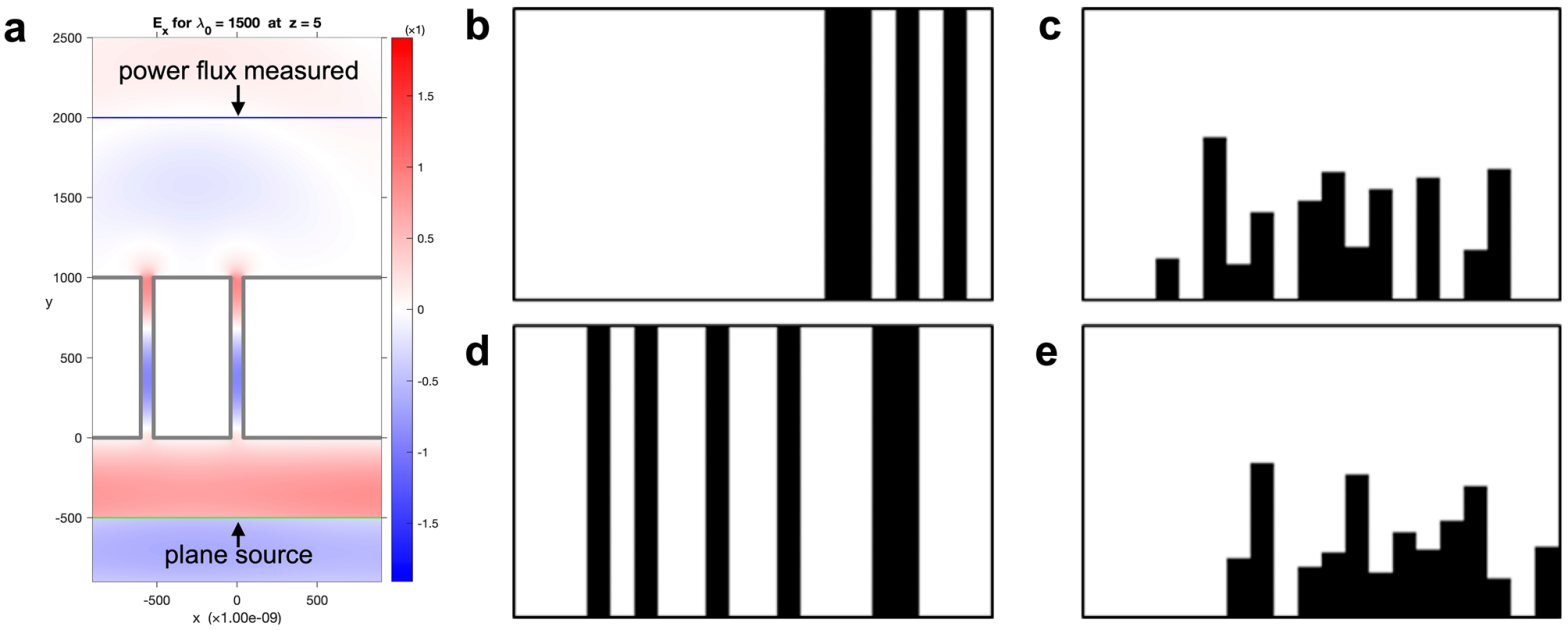
Example output of an open-source simulator and example images of the dataset. An open-source simulator called MaxwellFDFD is used to generate the designs of device images in the dataset. The transmittance is calculated at the y = 2000 plane, and the source is simulated at the y = -500 plane (**a**). The example images of the dataset include simple random data Types 1 (**b**) and (**d**), and complicated random data Types 2 (**c**) and (**e**). The black parts of the images represent the object material of simulation, and the white parts represents vacancy.

Images of object material of the simulation were generated from random numbers ranging from 0 to 2^20^ − 1. We generated the images by considering a 200 × 100-pixel image as 20 bars and replacing the random number with a binary number, as shown in Fig. 3b, d. For example, if “106” is the random number, “1101010” becomes the converted binary number, and the image shown in Fig. 3b is generated. Here, the black part represents “1” and the white part represents “0”. Since the object size of the MaxwellFDFD simulator is composed of approximately 2000 nm on the x-axis and 1000 nm on the y-axis by default, we scaled our image to 2:1. Also the pixel size was determined to be 200 × 100 similar to ImageNet^2^, a typical image classification challenge which includes 256 × 256-pixel images as dataset. As seen in Fig. 3c, e, we randomly adjusted the length of each bar to create a more complex and realistic structure. We defined as complicated design because more various designs are generated when the length of the objects in each position is determined at random as well as the position of the object. The reason for fixing the structure bottom is to make the structure feasible when cutting the nanomaterials with a laser.

After the images were generated randomly, we performed simulation by inserting objects of the same design as random images into the MaxwellFDFD simulator. Figure 3a demonstrates the visualisation result of the simulation. As a result of the simulation, the transmittance could be obtained by solving the frequency-domain form of Maxwell’s equations. We briefly review Maxwell’s equations and the process of calculating transmittance, for more information, see MaxwellFDFD^21^. Maxwell’s equations are written as:

$$\nabla \times E\left( r \right) = - i\omega \mu \left( {r,\omega } \right)H\left( r \right) - M\left( r \right),$$

$$\nabla \times H\left( r \right) = i\omega \varepsilon \left( {r,\omega } \right)E\left( r \right) + J\left( r \right),$$

where ${ }E\left( r \right), H\left( r \right)$ are electric and magnetic fields at a given point $r$, $J\left( r \right), M\left( r \right)$ are electric and magnetic current source densities at a given point $r$. $\varepsilon \left( {r,\omega } \right), \mu \left( {r,\omega } \right)$ are the electric permittivity and magnetic permeability of the object at a given point $r$ and a given oscillation frequency $\omega$. We used the background material as vacuum and the object material as silver, which is defined as ‘CRC/Ag’ in the simulator. After $E\left( r \right), H\left( r \right)$ are calculated through the equations, the power flux is then calculated from calculating the Poynting’s vector, which is defined as $S\left( r \right) = E\left( r \right) \times H\left( r \right)$. Specifically, we consider transmittance at wavelength *T*_*λ*_, which is given as *T*_*λ*_ = *Φ*_*e,λ*_^*t*^/*Φ*_*e,λ*_^*i*^ ≈ *P*_*λ*_^*obj*^/*P*_*λ*_^0^.* Φ*_*e,λ*_^*t*^ and *Φ*_*e,λ*_^*i*^ represent the radiant flux in the wavelength transmitted and received by the surface, respectively. *P*_*λ*_^*obj*^ and *P*_*λ*_^0^ represent the calculated power flux with a specific shape of the object and without any object in the simulation domain, respectively. Originally, the transmittance is calculated as the ratio of the radiant flux of the wavelength transmitted by the surface to the radiant flux of the received wavelength. However, as shown on the right side of the equation, transmittance is calculated as the ratio of the calculated power flux with a specific shape of the object to the calculated power flux without any object in the simulation domain.

We used the plane source at y = -500 nm and calculated the transmittance at y = 2000 nm, as illustrated in Fig. 3a. The simulation was performed by adjusting the wavelength of the plane source from 400 to 1,550 nm in units of 50 nm. As a result, we created a dataset that yields 24 output transmittances, having a float value between 0 and 1, per input image.

To train the prediction model, we generated 45,000 datasets, each of which was randomly split into 27,000, 9,000, and 9,000 images for train, validation, and test sets, respectively (Supplementary Table S1). We used the train set for model training, validation set for hyperparameter tuning, and test set for performance evaluation. We named the simple design of data in Fig. 3b, d as “Type 1” and a more complicated design of the data in Fig. 3c, e as “Type 2”. The reason why we created more data for Type 2 than Type 1 is that the shape of Type 2 is more complicated and more practical for real-time application. This imbalance in the number of different data types explains why the prediction results differ depending on the data type.

### Training model

Unlike fully connected neural networks (ANN) and other machine learning algorithms, CNNs achieve better generalisation on image data. It takes advantage of the spatial locality by applying local connectivity patterns between neighbouring neurons. In addition, in CNN, each filter is replicated across the entire visual field. These replicated units share the same weights and biases and form feature maps. This weight-sharing significantly reduces the number of available parameters learned, lowering the memory requirements for running the network and allowing much less training data.

**Figure 4:**
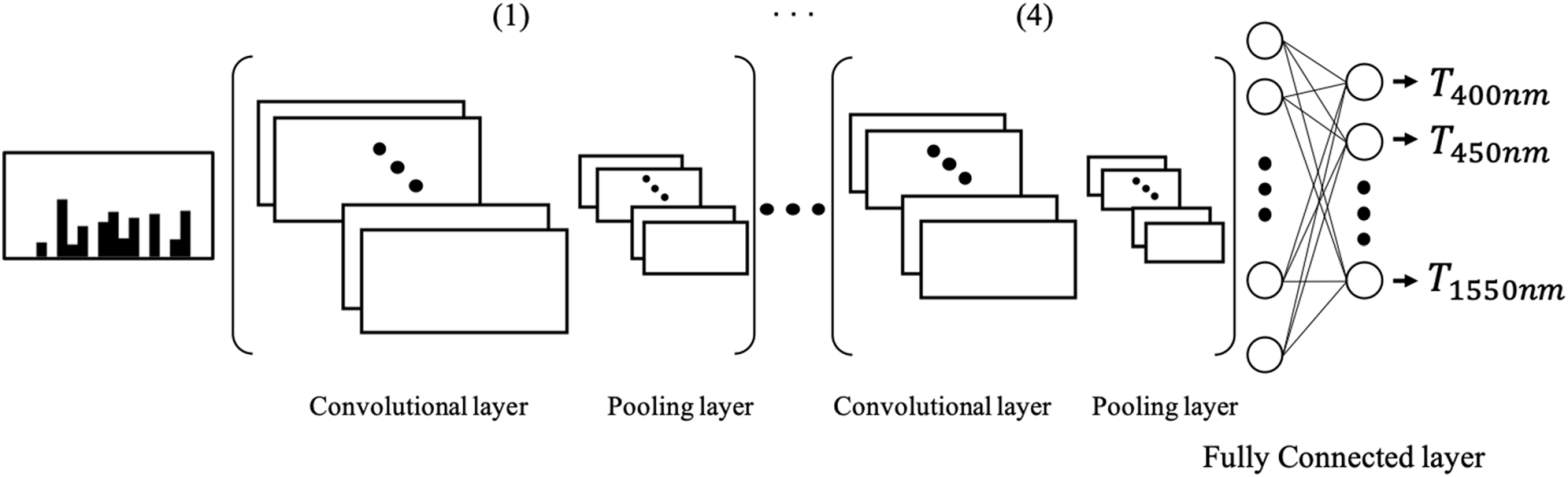
CNN architecture of the prediction model. We apply four sets of convolutional layers and pooling layers. In each layer, ReLU is used for the activation function after the 3 × 3 convolutional layer, and 2 × 2 MaxPooling is used to reduce the sizes of the images. The two fully connected layers have 1,024 and 24 neurons, respectively.

The CNN used in our study includes four convolutional layers, each followed by a pooling layer, which are then followed by two fully connected layers, as shown in Fig. 4. The number of channels in the first convolutional layer is 16 and the kernel size is 3 × 3. The other convolutional layers have 32 channels, again with the kernel size of 3 × 3. The validation results in terms of the number of channels and layers are shown in Supplementary Table S2. The model with four convolutional layers starting with a layer of 16 channels performed the best in validation set as well as in training set. In the pooling layers, the max-pooling function and a kernel size of 2 × 2 were adopted. The two fully connected layers had 1,024 and 24 neurons. The sigmoid function was adopted at the end of the fully connected layer, in order to obtain 24 regression results between 0 and 1 for an input image. We used the RMSE as the loss function and trained the batch size of 128 and 300 epochs. To prevent overfitting, we applied a dropout rate of 0.4 to the first fully connected layer. The validation results in terms of the dropout rate are shown in Supplementary Table S3. Because the training dataset was small and the images used were binary, we designed the number of CNN layers to be shallower and used fewer channels than most other CNNs, as seen in Supplementary Figure S1.

We trained other machine learning models to verify CNN’s performance, as well as a MLP comprising three layers with 512, 256, and 24 neurons. We also trained models based on tree models such as random forest (the number of estimators = 100, max depth = 30), decision tree (max depth = 5), and extra tree (the number of estimators = 10, max features = 32). The hyperparameters were tuned using a grid search, which is an algorithm that feeds the model a set of hyperparameters, and then runs an exhaustive search over all possible combinations of these values. The MLP and CNN models used 200 × 100 as the input image size, while the other models used 20 × 10 due to limited memory.

### Evaluating the performance

To evaluate the performance of the prediction models, we measured the RMSE and R^2^ scores, which are defined as follows: RMSE = $\sqrt {\frac{{\mathop \sum \nolimits_{i}^{n} \left( {\widehat{{T_{i} }} - T_{i} } \right)^{2} }}{n}}$, $R^{2} = 1 - \frac{{\mathop \sum \nolimits_{i} \left( {\widehat{{T_{i} }} - T_{i} } \right)^{2} }}{{\mathop \sum \nolimits_{i} \left( {T_{i} - \overline{T}_{i} } \right)^{2} }}$. The terms $\widehat{{T_{i} }}$ and $T_{i}$ denote the transmittance from the prediction models and MaxwellFDFD simulation, respectively, and the term $\overline{T}_{i}$ represents the average of MaxwellFDFD simulation results.

### Developing a model with practical performance

It is important to accurately predict the transmittance at each wavelength, but knowing which wavelengths have the highest and lowest transmittance is important in real design. To achieve this goal, we decided to include the differential value of the transmittance of adjacent wavelengths in the loss function in the following two ways.

$$dT_{i} = T_{i} - T_{i - 1} ,$$

$${\text{RMSE }} + {\text{ diff}}.{\text{ RMSE }} = \sqrt {\frac{{\mathop \sum \nolimits_{i}^{n} \left( {\widehat{{T_{i} }} - T_{i} } \right)^{2} }}{n}} + \sqrt {\frac{{\mathop \sum \nolimits_{i}^{n} \left( {\widehat{{dT_{i} }} - dT_{i} } \right)^{2} }}{n}} ,$$

$$y = \left\{ {\begin{array}{*{20}c} { 1} \\ { 0} \\ \end{array} } \right. \begin{array}{*{20}c} {if \left( {T_{i} - T_{i - 1} } \right) > 0} \\ {otherwise,} \\ \end{array}$$

$${\text{RMSE }} + {\text{ diff}}.{\text{ BCE }} = \sqrt {\frac{{\mathop \sum \nolimits_{i}^{n} \left( {\widehat{{T_{i} }} - T_{i} } \right)^{2} }}{n}} + \frac{{ - \mathop \sum \nolimits_{i}^{n} y_{i} \log \left( {\widehat{{y_{i} }}} \right) + \left( {1 - y_{i} } \right) \cdot {\log}\left( {1 - \widehat{{y_{i} }}} \right)}}{n}.$$

First, the diff. RMSE was added to the original RMSE. Second, we added the diff. BCE to the original RMSE. We calculated binary cross entropy loss, a function which measures how far away from the true value the prediction is for each of the classes and then averages the errors to obtain the final loss, as shown in the equation above. In this problem, there lies only two classes for each differential value of the transmittance, which is either increase or decrease (1 or 0 expressed by y). Therefore, we used binary cross entropy as a part of loss function rather than the categorical cross entropy originally proposed. To confirm the practical performance in numbers, we calculated the RMSE of the local maxima and minima, which are the locally largest and smallest values of transmittance in a domain of 24 wavelengths.

## Supplementary information

Supplementary information

## Data Availability

The dataset and source codes for this work are publicly available at https://github.com/wonderit/maxwellfdfd-ai
