# Supplementary material for: Simulation acceleration for transmittance of electromagnetic waves in 2D slit arrays using deep learning
Source: Sci Rep. 2020 Jun 29;10:10535. doi: 10.1038/s41598-020-67545-x (PMC7324378; doi:10.1038/s41598-020-67545-x)
Supplement: Supplementary file 1 — Supplementary information [file 41598_2020_67545_MOESM1_ESM.pdf]

Supplementary Materials

Simulation acceleration for transmittance of electromagnetic waves in 2D slit arrays using deep learning

Wonsuk Kim[1], Junhee Seok[1*]

[1]School of Electrical Engineering, Korea University, Seoul, 02841, Korea

[*]Corresponding author

Emails:

WK: won425@korea.ac.kr

JS: jseok14@korea.ac.kr

Data Type	Train	Validation	Test
Type 1	9,000	3,000	3,000
Type 2	18,000	6,000	6,000
Total	27,000	9,000	9,000

Table S1. The separation of the dataset. The dataset is randomly split into 27000, 9000, and 9000 images for train, validation, and test, respectively. We created more Type 2 images than Type 1, because Type 2 is more complicated and practical design.

Model<br>Hidden Layer Channels	RMSE		R^2 score	
	Train	Validation	Train	Validation
[16, 32]	0.0349	0.0814	0.9545	0.7603
[32, 32]	0.0340	0.0809	0.9570	0.7630
[16, 32, 32]	0.0315	0.0724	0.9637	0.8116
[32, 32, 32]	0.0313	0.0717	0.9640	0.8148
**[16, 32, 32, 32]**	**0.0310**	**0.0694**	**0.9645**	**0.8267**
[32, 32, 32, 32]	0.0362	0.0738	0.9499	0.8045
[16, 32, 32, 32, 32]	0.0347	0.0706	0.9580	0.8206
[32, 32, 32, 32, 32]	0.0342	0.0704	0.9592	0.8213
[16, 32, 32, 32, 32, 32]	0.0445	0.0727	0.9377	0.8098
[32, 32, 32, 32, 32, 32]	0.0434	0.0728	0.9413	0.8093

Table S2. Validation performance of CNN models in terms of the number of channels and layers. As the number of layer and channel increases, the error generally decreases and then increases again in specific parameters. The model with 4 layers using 16 channels in the first layer and 32 channels in the remaining layers showed best performance in the validation set.

Model Dropout Rate	RMSE		R^2 score	
	Train	Validation	Train	Validation
0	**0.0310**	0.0694	**0.9645**	0.8267
0.1	0.0326	0.0646	0.9605	0.8492
0.2	0.0335	0.0633	0.9580	0.8553
0.3	0.0347	0.0630	0.9546	0.8568
**0.4**	0.0364	**0.0623**	0.9496	**0.8594**
0.5	0.0387	0.0627	0.9426	0.8578

Table S3. Validation performance of CNN model in terms of the dropout rate. When the dropout was not used, the model was overfitted and the error of the training set was the lowest. When the dropout rate was 0.4, the performance of the validation set showed the best.

Model	Test set		Local maxima and minima of Test set	
	RMSE	R^2 score	RMSE	R^2 score
1	0.0629	0.8551	0.0702	0.8768
1 + 2	0.0600	0.8683	0.0676	0.8858
1 + 2 + 3	0.0588	0.8736	0.0666	0.8892
1 + 2 + 3 + 4	**0.0584**	**0.8754**	**0.0662**	**0.8907**

(1: RMSE, 2: MSE, 3: RMSE + diff. BCE, 4: RMSE + diff. RMSE)

Table S4. Model performance of ensemble models. The number of models refers to the model trained with the specific loss function listed below the table. The ensemble model is generated by calculating the average of each model's output.

Model	Number of images (Train / Test)	Test set	Local maxima and minima of Test set
		RMSE	RMSE
Type 1	9,000 / 3,000	0.0748	0.0771
Type 2-half	9,000 / 3,000	0.0735	0.0834
Type 2	18,000 / 6,000	**0.0560**	**0.0654**

Table S5. Model performance of CNN model in terms of the composition of number and type of dataset. The model ‘Type 1’ refers to the model trained and tested with Type 1 dataset. The model which name starts with ‘Type 2’ refers to the model trained and tested with Type 2 dataset. The model ‘Type 2-half’ refers to the model trained with the same number of datasets as the model ‘Type 1’.

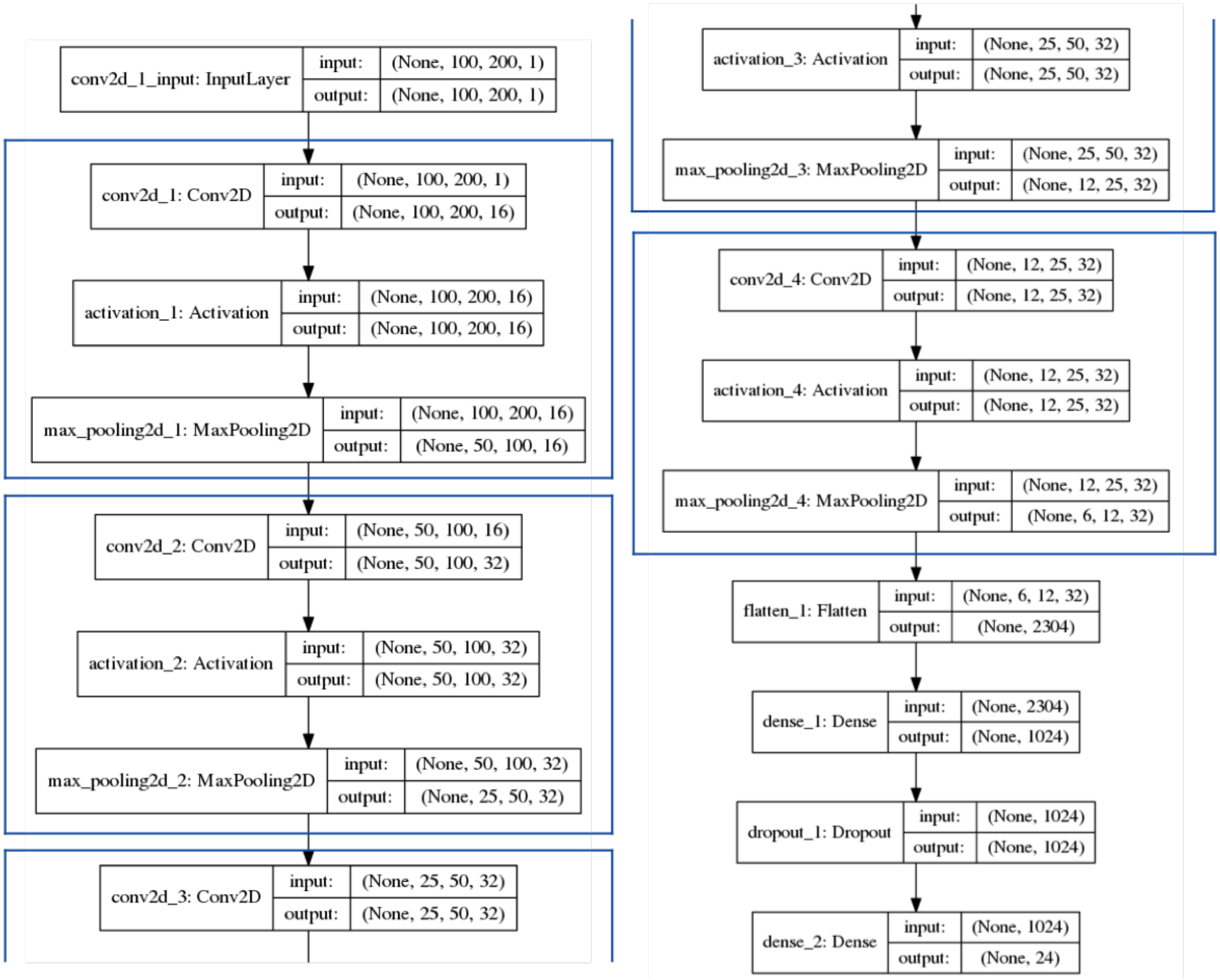

Figure S1. Model layout for the CNN model. The model consists of 4 sets of convolutional layer and polling layer. The input image is 200 pixels wide and 100 pixels high. The two fully connected layers at the end have 1024 and 24 neurons, respectively.

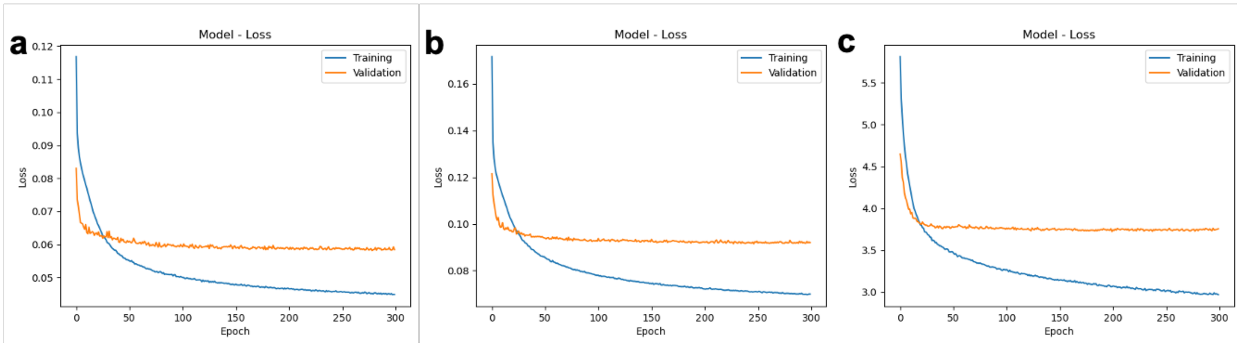

Figure S2. The training and validation curves in terms of the composition of the loss function. Learning curve of the (a) model trained by loss function of RMSE, (b) model trained by the loss function that adds the RMSE of the differential to the RMSE, and (c) model trained by the loss function that adds the BCE of the differential to the RMSE.

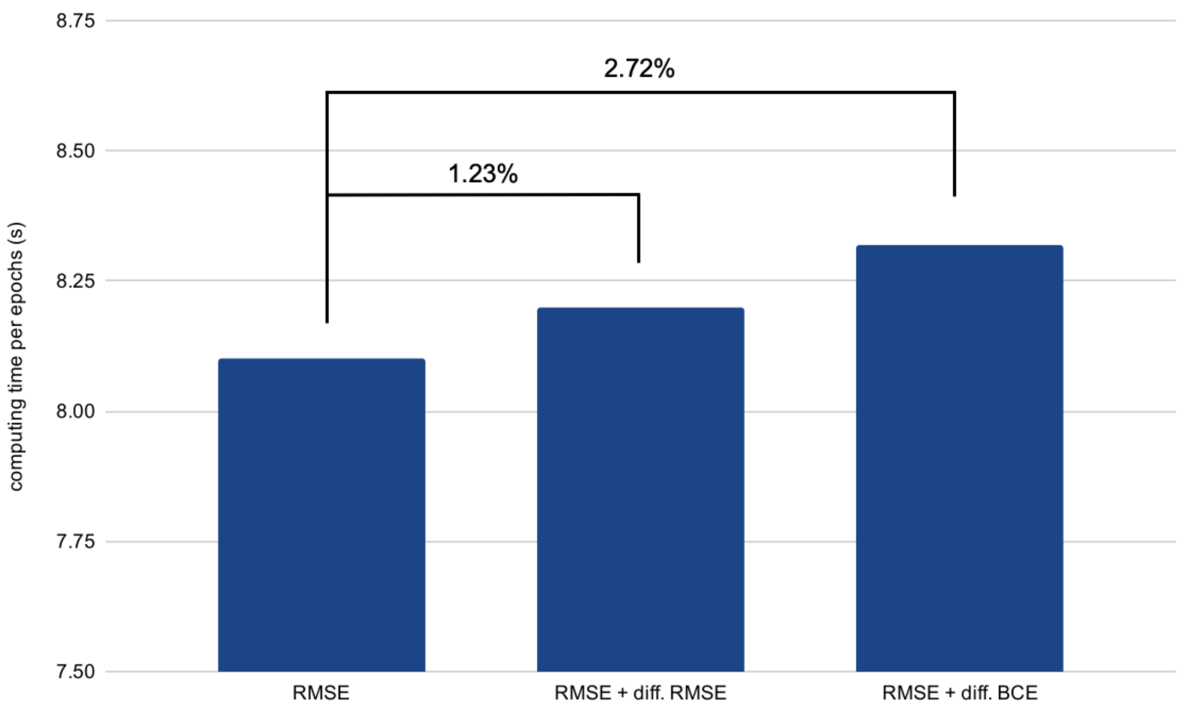

Figure S3. Comparison of computational time between the CNN models in terms of the composition of the loss function. The computational time was measured per epochs during training process using an NVIDIA GTX 1080Ti GPU and a single 4-core CPU. The difference in calculation time ranged from 0.1 to 0.22 seconds per epoch, which is about 32 to 66 seconds when performing 300 epochs.
